# Families stuck in a trauma loop: a systemic approach to address vicarious trauma and enhance mental health treatment adherence in 2nd and 3rd generation offsprings of psychiatric patients

**DOI:** 10.1192/j.eurpsy.2025.2139

**Published:** 2025-08-26

**Authors:** S. Andrei, B. Raz

**Affiliations:** 1 Psychiatry and Psychotherapy, Private Practice, Challans; 2 Family Therapy Center, CH Roanne, Roanne, France

## Abstract

**Introduction:**

Vicarious trauma (VT) was first described by Pearlman and McCann as the therapists’ internal experience that is “impacted as a result of empathic engagement with client’s traumatic material”. (JTS 1990. 3: 131–149). Furthermore, the authors used a constructivist framework to differentiate it from other forms of secondary trauma. In the context of mental illness, family elements of VT are be more subtle than primary trauma, but can remain identifiable across generations.

**Objectives:**

The primary focus of our study was to develop a theoretical framework for understanding severe mental illness (SMI) and its traumatic long-term consequences on the family. Our secondary aim was to explore the impact of SMI and of psychiatric stigma on 2nd and 3rd generation offsprings.

**Methods:**

To begin with we conducted a narrative review of the scientific literature using Pubmed search engine. This was followed by a qualitative study with psychiatric patients who declared a family history of SMI in a first degree relative who received medical care in France. The methodology involved conducting 11 structured interviews, which were audio recorded, transcribed and thematically analyzed.

**Results:**

When family trauma develops, it is a complex phenomenon, seemingly infinite loop. It further impacts every aspect of the family’s communication and relational style, attachment and regulation.

On an individual level, the consequences can be viewed as a continuum from adverse childhood experiences and primary trauma to secondary traumatic responses. This study aims to advocate for identifying specific VT subtypes. Some specific complaints are often encountered in the initial presentation of psychiatric patients, even with adults as young as 18yo. Exploring family psychiatric history through the vicarious “trauma lens”, can allow us to have a better appreciation and understanding of the patient’s suffering: their system of meaning and beliefs regarding psychiatric diagnosis, treatment and case management are often disrupted, the nocebo effect is increased (by increased sensitization to all other trauma and by modified emotion control). When the sense of safety is disturbed on a family basis multi-generational level, the consequences could lead to extreme anti-psychiatry attitudes and predict negative mental health outcomes.

**Conclusions:**

Current understanding of vicarious trauma is limited to specific job-related contexts. What has not been studied yet is the vulnerability of children to vicarious trauma due to perceived traumatizing treatment, or the way in which medical stories are recounted to children. Therefore, additional research is needed on this topic. For psychiatrists, the vicarious trauma approach can be one of the key entry points to building the therapeutic alliance, which further enhances patient adherence to the treatment.

**Disclosure of Interest:**

None Declared

